# Behavioural oscillations in visual orientation discrimination reveal distinct modulation rates for both sensitivity and response bias

**DOI:** 10.1038/s41598-018-37918-4

**Published:** 2019-02-04

**Authors:** Huihui Zhang, Maria Concetta Morrone, David Alais

**Affiliations:** 1 School of Psychology, University of Sydney, Sydney, 2006 New South Wales Australia; 2 Department of Translational Research on New Technologies in Medicine and Surgery, University of Pisa, 56123 Pisa, Italy; 3 Scientific Institute Stella Maris, 56018 Calambrone, Pisa Italy

## Abstract

Perception is modulated by ongoing brain oscillations. Psychophysical studies show a voluntary action can synchronize oscillations, producing rhythmical fluctuations of visual contrast sensitivity. We used signal detection to examine whether voluntary action could also synchronize oscillations in decision criterion, and whether that was due to the oscillations of perceptual bias or of motor bias. Trials started with a voluntary button-press. After variable time lags, a grating at threshold contrast was presented briefly and participants discriminated its orientation (45° or −45°) with a mouse-click. Two groups of participants completed the experiment with opposite mappings between grating orientations and response buttons. We calculated sensitivity and criterion in the 800 ms period following the button press. To test for oscillations, we fitted first-order Fourier series to these time series. Alpha oscillations occurred in both sensitivity and criterion at different frequencies: ~8 Hz (sensitivity) and ~10 Hz (criterion). Sensitivity oscillations had the same phase for both stimulus-response mappings. Criterion oscillations, however, showed a strong anti-phase relationship when the two groups were compared, suggesting a motor bias rather than perceptual bias. Our findings suggest two roles for alpha oscillations: in sensitivity, reflecting rhythmic attentional inhibition, and in criterion, indicating dynamic motor-related anticipation or preparation.

## Introduction

Recent neurophysiological studies show that ongoing oscillatory brain activity just prior to an incoming stimulus is critical in shaping subsequent perception. Increased power in pre-stimulus alpha oscillations (8–12 Hz) is linked to poorer visual detection performance^1–4^ and greater pre-stimulus theta (4–8 Hz) power is associated with better memory encoding^5,6^ and retrieval^7^. Phase is important too, with the phase of pre-stimulus theta or alpha oscillations predicting subsequent visual detection^8–10^. Several groups have shown that phase can be manipulated using a salient visual or auditory event to reset the phase of ongoing oscillations and align them with that event^11–14^.

Psychophysical studies probing visual performance following a phase-resetting event reveal perceptual oscillations in the theta/alpha range^15–20^. Landau and Fries used this approach to show that visual attention samples information rhythmically following a brief flash at one of two locations^15^. Performance at each location oscillated at 4 Hz, but in antiphase, consistent with an 8 Hz sampling process alternating between locations^21^. Other studies have used cross-modal stimuli^22^ or voluntary action^19,20^ to reset phase and induce rhythmical visual performance. Together, these studies suggest that perception is not determined solely by sensory stimuli but also by the phase of ongoing neural oscillations.

Perceptual performance, according to Signal Detection Theory (SDT), is a combination of sensitivity to sensory stimuli and decision criterion^23–25^. According to SDT, criterion can be set and changed by the observer, while sensitivity cannot. One proposed function for alpha oscillations is in top-down control through active suppression of irrelevant information^26–28^ and, consistent with this, neuronal recordings suggest that alpha waves propagate in a feedback direction^29^. Alpha oscillations may therefore be expected to influence criterion more than sensitivity, although separating these factors was not possible in earlier studies as their designs contained only target-present trials. In an EEG study, Sherman *et al*. resolved this problem and found occipital alpha phase influenced the decision criterion rather than the sensitivity of visual detection^30^, consistent with alpha oscillations reflecting rhythmic top-down expectations. It is possible that oscillations might influence sensitivity too, but at a different frequency, and a recent study by Ho *et al*. found behavioural oscillations in both sensitivity and criterion – at different frequencies – in an auditory task^31^.

The present study uses an SDT design to examine whether oscillations of sensitivity and decision criterion occur in a visual discrimination task. We follow previous studies and use voluntary action as a phase resetting tool to induce rhythmic modulations of visual contrast sensitivity^19,20^ and to test whether voluntary action also induces oscillations of criterion. Participants initiate each trial by a voluntary button-press and then discriminate the orientation of a brief foveal grating presented after a random interval drawn from a finely sampled range following the button-press. An SDT analysis will examine if voluntary action induces periodic oscillations of sensitivity and/or criterion. A second aim is to clarify whether any observed criterion oscillation is driven by perceptual bias or motor bias, two biases that are usually confounded in most experimental designs^32^. Previous studies have revealed that pre-stimulus alpha oscillations can occur over occipital^30^ or motor cortex^33^ and argued that they reflect prior expectations. Here we decouple perceptual and motor biases by comparing two groups who used opposite mappings between the grating orientations and response buttons. If the change in response-mappings alters the criterion oscillation, it would indicate a motor-related source rather than a perceptual one.

## Results

**Figure 1:**
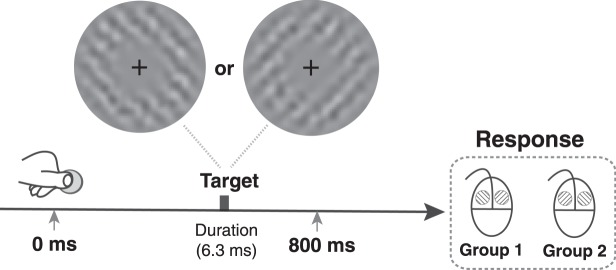
Illustration of the procedure. Participants pressed a button to start each trial (voluntarily self-initated). A brief target (grating embedded in noise) was presented for 6.3 ms after a variable delay (0–800 ms). The contrast of the grating was varied to maintain threshold level performance. Participants were required to respond on a two-button mouse which orientation (clockwise or anti-clockwise) they perceived. Two groups were tested, each using a different mapping between stimulus orientation and response button, as illustrated.

The task was to discriminate the grating orientation (45° or −45°) at threshold contrast level (75% correct) after a voluntary button press (Fig. 1). Two groups of participants used a mouse to report their answers. Participants in Group 1 clicked the left to report ‘anticlockwise’ orientation, and the right for ‘clockwise’ orientation. The mapping between orientations and response buttons was reversed for participants in Group 2. Note, the response was not bimanual. Although the response mapping was switched for two groups, the switch was within a single hand’s digits (see Fig. 1). All the participants attended two sessions over two days, each of which consisted of three blocks. We first analysed the threshold contrast of grating across six blocks. Two participants were excluded from further data analysis: one participant had high discrimination threshold (more than three standard deviations from the mean) in the first block (contrast 7.6%) and the other had high threshold in the fourth, fifth, and six blocks (7.4%, 6.8%, and 8.7%). We analysed the data from the remaining 29 participants (contrast 4.5% ± 0.5%), 15 for Group 1 and 14 for Group 2. For each of the remaining participants, we eliminated trials with reaction times more than three standard deviations away from the mean of reaction times.

The data of all 29 participants from Group 1 and Group 2 were pooled together as an aggregated observer. To examine the fluctuation of the aggregated observer’s performances over time, we binned the data every 15 ms (53 data points in the range of 5–800 ms). According to SDT, both sensitivity and criterion determine detection or discrimination performance, although whether the criterion reflects perceptual bias or motor response bias often remains unknown because perceptual choices and behavioural responses are usually inseparable on a given task. Here, two groups of participants were compared who had opposite mappings between grating orientation and response button, allowing us to separate perceptual bias and motor bias.

**Figure 2:**
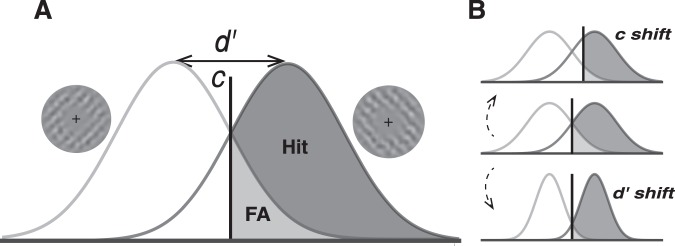
The signal detection theory (SDT) analysis. (**A**) Two probability distributions corresponding to distributions of a decision-maker’s internal response for clockwise orientation and anticlockwise orientation. The sensitivity (d′) is dertermined by the seperation between these two distributions. The vertical line indicates criterion (c). For analysis based on stimulus, we chose anticlockwise condition (trials with anti-clockwise grating for both Group 1&2) to calculate the hit rate (Hit) and clockwise condition to calculate false alarm rate (FA).The decision-maker reports ‘anticlockwise’ if the internal response is greater than c. For analysis based on response, we chose the ‘left-click’ conditions (trials presenting anticlockwise grating in Group 1, and clockwise grating in Group 2) to calculate the Hit rate. The False Alarm rate was acquired by the percentage of ‘left-click’ in trials where the right answer was ‘right-click’ (trials presenting clockwise grating in Group 1, and anticlockwise grating in Group 2). (**B**) The illustration of criterion and sensitivity shifts.

In the framework of SDT (Fig. 2), we analysed the data in two ways to obtain criterion measures, one based on stimulus and one based on response (see Methods), to separate the effects of perceptual bias and motor response. *Stimulus-based analysis*: we chose ‘anti-clockwise’ choices to calculate Hit rate and ‘clockwise’ choices to calculate False Alarm rate (the order is arbitrary in a two-alternative SDT analysis). Hit rate was therefore the percentage of ‘anti-clockwise’ choices (left clicks in Group 1, and right clicks in Group 2) in trials presenting an anti-clockwise grating, while False Alarm rate was the percentage of ‘anti-clockwise’ choices in trials presenting a ‘clockwise’ grating. For the data aggregated over both groups, this meant calculating perceptual choices regardless of left or right mouse clicks, depending on which group the data came from. Thus, for the aggregated observer, the criterion acquired with the stimulus-based analysis reflects perceptual (stimulus-driven) bias. *Response-based analysis*: we used ‘left-click’ response to calculate Hit rate and ‘right-click’ response to calculate False Alarm rate. The Hit rate is given by the ‘left-click’ percentage in trials where the correct answer was ‘left-click’ (trials presenting anticlockwise grating in Group 1, and clockwise grating in Group 2), while the False Alarm rate is acquired by the ‘left-click’ percentage in trials where the correct answer was ‘right-click’ (trials presenting clockwise grating in Group 1, and anticlockwise grating in Group 2). Thus, for the aggregated observer, the criterion acquired with the response-based analysis reflects motor bias; the sensitivity measure should be invariant from the response mapping.

### Analysis of Groups 1 and 2 combined

**Figure 3:**
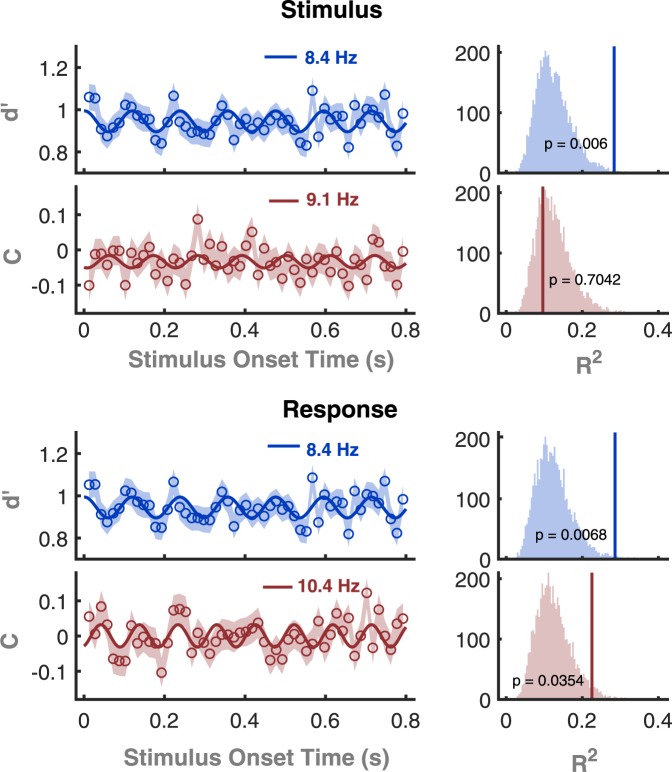
(**A**) Data from Groups 1 and 2 pooled into a single aggregated observer showing performance over time on the two dependent measures (sensitivity and criterion) with anlaysis based on Stimulus (**A**) or Response (**B**). Left: the open symbols are the real data points, with ±1 SEM (shaded area) obtained from bootstrapping, at intervals of 15 ms. The continuous line is the best-fitting curve from the Fourier series. Right: Results from the permutation test (n = 5000). The histogram shows the distribution of R^2^-values obtained from fitting the Fourier series to the permutated data. The red and blue vertical lines show the R^2^ of the best-fitting Fourier series to the real data, with its p-value labelled.

For the aggregated observer, we calculated sensitivity and criterion at each time point using these two ways (stimulus-based and response-based) of analysis. We fitted first-order Fourier series in the theta-alpha range (3–13 Hz) to these time series and ran permutation tests (n = 5000) to examine the significance of frequency of greatest R^2^ (Methods). For sensitivity, significant oscillations were found with both analysis methods. The best-fit was obtained at 8.4 Hz, for both the stimulus-based analysis (R^2^ = 0.28, permutation test, p = 0.006, Fig. 3A) and the response-based analysis (R^2^ = 0.29, permutation test, p = 0.0068, Fig. 3B). For criterion, however, a significant oscillation was found only with the response-based analysis. The best-fit with the response-based analysis was at 10.4 Hz (R^2^ = 0.23, permutation test, p = 0.0354, Fig. 3B) and the stimulus-based analysis yielded a non-significant best fit at 9.1 Hz (R^2^ = 0.096, permutation test, p = 0.7042, Fig. 3A).

We also calculated the sensitivity and criterion for each individual participant, using the same 15-ms time points, and acquired the group average. We fitted first-order Fourier series in the same theta-alpha range (3–13 Hz) to the time series of the group mean data and ran permutation tests (n = 5000, for each permutation, the responses were shuffled across trials within each participant and then averaged across participants) to examine the significance of frequency of greatest R^2^. The results for best-fitting frequency were identical to those acquired from analysis of the aggregated data (see Supplementary Fig. S1).

The absence of a significant criterion oscillation with the stimulus-based analysis is important. It suggests that voluntary action could effectively synchronize alpha oscillations in sensitivity and in response (motor) bias, but not in perceptual bias. Moreover, the oscillation of sensitivity was unaffected by the manipulation of motor responses, indicating that perceptual processing occurs independently of motor-related activity.

**Figure 4:**
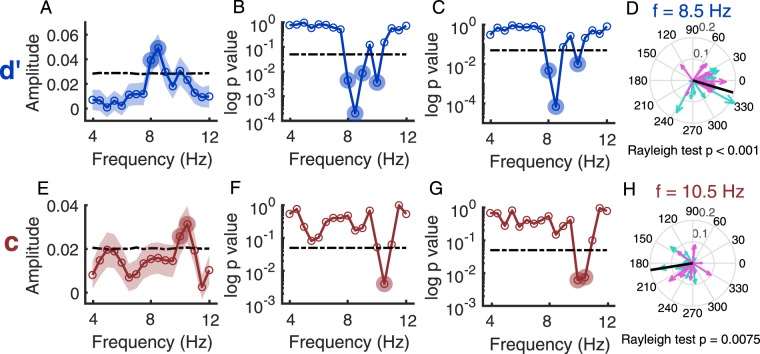
Data showing spectral analyses for sensitivity (top row) and criterion-motor bias (bottom row). A & E: Amplitude spectrum of the aggregated data (both groups, n = 29) in the range of 4–12 Hz at steps of 0.5 Hz. The black dash-dot line indicates the 95th percentile threshold of amplitudes from permutated data at each frequency. Open circles represent the data points at different frequencies. The filled circles indicate significant frequencies after FDR (10%) correction. B & F: The result of permutation Hotelling’s T-square test at each frequency. P-values are shown on a log scale. The black horizontal dash-dot line indicates p = 0.05. The filled circles indicate significant frequencies after FDR (10%) correction. C & G: Phase consistency among the 29 observers measured at each frequency using the Rayleigh test. P-values are shown on a log scale. The black horizontal line indicates p = 0.05. The filled circles indicate significant frequencies after FDR (10%) correction. D & H: Polar plots showing phases at the frequency of greatest amplitude for the aggregated (black line) and individual participants (cyan arrows: Group 1; magenta arrows, Group 2).

To further examine the oscillation frequency spectrum, we fitted the first-order Fourier series in steps of 0.5 Hz from 4 to 12 Hz to the time series of sensitivity and criterion obtained from the response-based analysis for the aggregated data (Fig. 4). We ran permutation tests (n = 5000) to examine the significance of spectrum amplitude at each frequency in the theta-alpha range (17 frequencies, 4–12 Hz). At each frequency under test, we fitted first-order Fourier series at that frequency, with all other parameters free. We also performed multiple comparisons correction across all 17 frequencies using false discovery rate (FDR) of 10%^34^. For sensitivity, the spectral amplitude was significantly greater than that acquired from permutated data at 8.0 and 8.5 Hz (p < 0.05, FDR corrected), peaking at 8.5 Hz. For criterion obtained with the response-based analysis (i.e., motor bias), the amplitude was significant at 10 and 10.5 Hz (p < 0.05, FDR corrected), peaking at 10.5 Hz.

We also fitted the first-order Fourier series in steps of 0.5 Hz from 4 to 12 Hz (17 frequencies) to the time series of sensitivity and criterion obtained from the response-based analysis for each participant. For each frequency, we acquired a sample vector of cosine and sine components from fitting (see Methods). A second-level random-effect analysis was run to test whether the mean of these vectors (n = 29) were different from [0,0] at each of the 17 frequencies. Because Hotelling’s T-square test requires a multivariate normal distribution of samples, here we performed a permutation Hotelling’s T-square test (see Methods). After multiple comparisons correction (FDR = 10%), for sensitivity, the test was significant at 8.0, 8.5, 9, and 10 Hz, peaking at 8.5 Hz; for criterion obtained with the response-based analysis (i.e., motor bias), the amplitude was significant at 10.5 Hz (Fig. 4). The results from the random-effect analysis are consistent with the results acquired from the fixed-effect analysis on aggregated data.

Next, we examined phase consistency between participants. We obtained phase information at each frequency from each participant’s data and performed a Rayleigh test from the CircStats toolbox^35^. For sensitivity, phases of individual participants from both groups were clustered at 8, 8,5, and 10 Hz (p < 0.05, FDR corrected), which was consistent with the results of analysis on aggregated data and Hotelling’s T-square test. At the frequency of greatest amplitude (8.5 Hz), phases were clustered (p < 0.001, uncorrected) with a mean phase of −16.7°, 95% CI [−42.4°, 9.0°], similar to the phase obtained from the aggregated observer (−0.36°). The significant phase non-uniformity indicates consistency across individual participants at the frequency band corresponding to peak amplitude. For criterion obtained with the response-based analysis (motor bias), phases were clustered at 10 and 10.5 Hz (p < 0.05, FDR corrected), which is similar to the results from Hotelling’s T-square test and analysis on the aggregated data. At the frequency of greatest amplitude (10.5 Hz), phases were clustered (p = 0.0075, uncorrected) with a mean phase of −171.1°, 95% CI [−209.3°, −133.0°], similar to the phase of the aggregated observer (−154.8°).

The results from the fixed-effect analysis on the aggregated data, the second-level random-effect analysis for individual participants’ data, and the phase uniformity analysis were consistent, showing that visual sensitivity oscillated at ~8 Hz and criterion (motor bias) oscillated ~10 Hz. The oscillations were therefore not contributed by only a few participants but reflect a consistent behaviour across participants.

### Analysis of Group 1 vs. Group 2

**Figure 5:**
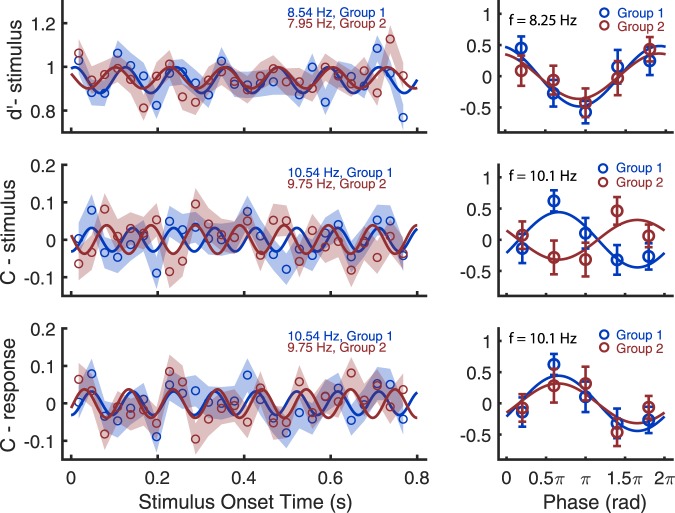
(**A**) Curve-fitting results for the data aggregated seperately over Group 1 (blue) and Group 2 (red). Left column: open symbols are the real data points and the continuous line is the best-fitting curve from the Fourier series. The shaded area indicates ± 1 SEM obtained from bootstrapping. Right column: The mean frequency of the two groups was used to epoch each individual’s data and combine their time series into a single period. The data within the period were divided into 5 phase bins, and then averaged across observers. This is shown for sensitivity in the top row (mean frequency = 8.25 Hz), and for criterion in the middle and lower rows (mean frequency = 10.1 Hz). The stimulus-based analysis (middle) produces a clear antiphase relationship when key mappings are reversed. All error bars indicate ±1 SEM.

To directly compare the two groups’ performance further, we analysed the data from the two groups separately. The data of all 15 participants in Group 1 were pooled together as an aggregated observer. We did the same for the 14 participants in Group 2. To reduce noise, we binned the data every 30 ms (26 data points in the range of 2.5–800 ms). For the two aggregated observers, we calculated sensitivity and criterion for each data point with SDT by using the stimulus-based analysis. For criterion, the DC bias was removed. We fitted first-order Fourier series in the theta-alpha range (3–13 Hz) to these time series. For sensitivity, the best-fits were obtained at ~8 Hz (Fig. 5, top-left): 8.54 Hz for Group 1 (R^2^ = 0.35), and 7.95 Hz for Group 2 (R^2^ = 0.27). Although the R^2^ values did not pass our strict permutation test in which permutated data were fit using Fourier series with frequency as one of the free parameters (p = 0.13 and p = 0.38 for Group 1 and Group 2, respectively), they were significantly greater than R^2^ values acquired from permutation when we fitted the permutated data using Fourier series with the same frequency acquired from real data (p = 0.006, and p = 0.03, respectively). Importantly, the frequencies were consistent with the frequency acquired from the data aggregated over both groups of participants and reported above. The sensitivity oscillations for each group seem to be in phase.

For criterion, the best-fits were at ~10 Hz (Fig. 5 middle-left): 10.54 Hz for Group 1 (R^2^ = 0.26), and 9.75 Hz for Group 2 (R^2^ = 0.30). Similar to sensitivity, the R^2^ values were not significant for the strict permutation test (p = 0.45, and p = 0.29, respectively), but they were greater than the R^2^ values acquired from permutation when fitting the permutated data using Fourier series with the same frequency acquired from the real data (p = 0.03 for Group1; p = 0.016 for Group 2). The frequencies we acquired for Group 1 and Group 2 were similar to the frequency acquired from the data aggregated over both groups using the response-based analysis (motor bias). The criterion oscillations acquired with stimulus-based analysis for the two groups appear to be in anti-phase. We also examined criterion with response-based analysis. For Group 1, it was the same as the criterion acquired with the stimulus-based analysis; for Group 2, the best-fit frequency was the same but it was in antiphase with the criterion acquired with the stimulus-based analysis. The criterion oscillations acquired with response-based analysis for the two groups appear to be in phase.

To examine more clearly the phase relationships between the two groups for both sensitivity and criterion, we epoched the data using the mean frequency of the two groups. That is, we divided the time series into epoch periods and averaged the data across epochs (Fig. 5, right-hand side). For each participant, we binned the time points according to their positions in a period of 8.25 Hz (mean of the best-fit frequencies in sensitivity) evenly into 5 bins for each participant and used SDT with the stimulus-based analysis to calculate sensitivity for each bin; we binned the time points into 5 bins according to their positions in a period of 10.1 Hz (mean of the best-fit frequencies in criterion) and used SDT with the stimulus-based analysis and response-based analysis to calculate criterion for each bin. Both sensitivity and criterion were transformed to z-scores for each participant across the 5 phase bins. We fitted the corresponding frequency (8.25 Hz for sensitivity and 10.1 Hz for criterion) to the mean of the epoched data for Group 1 and Group 2: for sensitivity, R^2^ = 0.85 for Group 1 and R^2^ = 0.86 for Group 2; for criterion, R^2^ = 0.83 for Group 1 and R^2^ = 0.63 for Group 2. For sensitivity, the phases for the two groups were in phase (13.6° and 14.9° for Group 1 and Group 2, respectively, 1.3° difference). For criterion obtained with stimulus-based analysis, the phases for the two groups were in anti-phase (−118.4° and 62.2° for Group 1 and Group 2, respectively, 180.6° difference). For criterion obtained with response-based analysis, however, the phases for two groups were in phase (−118.4° and −117.8° for Group 1 and Group 2, respectively, 0.6° difference).

We ran a two-way mixed ANOVA to examine the effect of group and phase bin on sensitivity and criterion. For sensitivity (Fig. 5, top right), there was no interaction between group and phase bin (F(4,108) = 0.518, p = 0.723, η_p_^2^ = 0.019) but there was a significant main effect of phase bin (F(4,108) = 3.672, p = 0.008, η_p_^2^ = 0.12). Multiple comparisons with Bonferroni correction revealed that sensitivity for the phase bin 3 (mean = −0.50) was significantly lower than that for phase bin 1 (mean = 0.27, 0.8 π distance from bin 3), and bin 5 (mean = 0.34, 0.8 π distance from bin 3), p = 0.030 and 0.011, respectively. For criterion acquired from stimulus-based analysis (Fig. 5, middle right), there was a significant interaction between group and phase bin (F(4,108) = 3.404, p = 0.012, η_p_^2^ = 0.112). When we broke down the interaction to examine the effect of phase bin on criterion for each group, there was no significant main effect of phase bin for Group 1 (F(4,56) = 2.437, p = 0.058, η_p_^2^ = 0.148) or Group 2 (F(4, 52) = 1.459, p = 0.228, η_p_^2^ = 0.101). This might be caused by low statistical power from the relatively small sample size. Next, we calculated the criterion from the response-based analysis (motor bias: Fig. 5, bottom right). The two-way mixed ANOVA revealed that there was no significant interaction between group and phase bin (F(4, 108) = 0.437, p = 0.781, η_p_^2^ = 0.016) but there was a significant main effect of phase bin (F(4,108) = 3.404, p = 0.012, η_p_^2^ = 0.112). Multiple comparisons with Bonferroni correction showed that criterion for phase bin 2 (mean = 0.453) and phase bin 4 (mean = −0.392, 0.8 π distance from bin 2) were different (p = 0.011). Together with the previous results on the aggregated observer over both groups of participants, our results show that voluntary action can reset the alpha oscillations in sensitivity (~8 Hz) and motor response bias (~10 Hz).

## Discussion

In the current study, we demonstrated that voluntary action synchronizes behavioural oscillations in the alpha band (8–12 Hz) for both sensitivity and criterion in an orientation discrimination task. Although sensitivity and criterion both oscillated in the alpha range, they did so at different frequencies (~8 Hz for sensitivity and ~10 Hz for criterion). Further, by using opposite mappings between grating orientations and response buttons for two groups of participants, we showed that the sensitivity oscillation was not affected by the reversal of response keys but criterion oscillation was. While it is not surprising that sensitivity – as an indicator of low-level sensory processing – was not affected by the manipulation of motor response, the criterion oscillation was susceptible to the mapping between stimulus and response and suggests that it was driven by the oscillation of motor bias rather than perceptual bias.

Consistent with previous findings^19,20^, we confirmed that voluntary action can synchronize ongoing oscillations in sensitivity. We found that the sensitivity oscillated in the low alpha range (~8 Hz) after the voluntary action. Moreover, changing the mapping between grating orientation and mouse clicks did not influence oscillation of sensitivity, which confirms that sensitivity reflects low-level sensory processing. Tomassini *et al*. showed that the oscillation of contrast sensitivity began before the execution of movement, suggesting oscillatory dynamics may mediate the automatic coupling between early motor planning and early visual processing^19,36^. Interestingly, for a same visual orientation discrimination task, Tomassini *et al*. found both theta (~4 Hz) and alpha (~10 Hz) oscillations^36^ corroborating the present results. Moreover, Tomassini and D’Ausilio^37^ found that stimulation of the median nerve, which activated the hand somatomotor system, triggered alpha oscillations (~10 Hz) in visual sensitivity. In contrast to low alpha oscillations we found here, Tomassini *et al*. and Benedetto *et al*. found visual perceptual periodicity in theta band locked to voluntary hand and finger movement^19,20^. The variability in frequency oscillation is very high across subjects and conditions as observed in many studies^16,19,20,31^. The origin of the variability is unclear, although it is known that task difficulty and visual stimuli properties, like overall luminance, may partly explain it^20,38^. Nevertheless, our results together with all these previous findings, demonstrate that voluntary action has a tight relationship with the perception of subsequent sensory events. It is possible that both endogenous intention to execute action and the exogenous activation of the sensorimotor system are both aligned with visual perception periodically.

Using a visual cue presented in the right visual field to synchronize endogenous oscillation, Landau and Fries found that after a flash was presented in the right visual field, subsequent detection performance at both the same and opposite locations oscillated at 4 Hz, but in antiphase^15^. Their findings could be explained by an attentional mechanism sampling at roughly 8 Hz. Because there were two locations sharing the same attentional sampling mechanism, this results in 4 Hz oscillations at both locations in antiphase as the sampling process alternates between locations. In our study, the two grating orientations were presented at a single (foveal) location and the oscillation at ~8 Hz in sensitivity we found here was in line with this explanation. Numerous studies have shown that alpha oscillations are related to functional inhibition^3,26–28,39–41^. Klimesch *et al*. proposed an inhibition-timing hypothesis of alpha rhythm, stating that alpha reflects top-down inhibitory and temporal control^26^. The oscillation of sensitivity around 8 Hz we found here is possibly subserved by this periodical inhibition and excitation of top-down attention.

The oscillations of sensitivity and criterion likely reflect different neural mechanisms. Two results supporting this conclusion are that, first, criterion and sensitivity oscillated at different frequencies, and second, the sensitivity oscillation was immune to the reversal of the mapping between stimuli and response buttons whereas the criterion oscillation was not. Electrophysiological studies have shown that sensitivity and criterion are related to activities in different brain regions: visual sensitivity in orientation change is reflected by the activity in V4^42^, whereas superior colliculus plays an important role in determining criterion of saccade target selection^43^. Sensitivity is a reflection of low-level sensory processing, while criterion is related to decision-level bias (perceptual bias, attentional bias, or motor bias). The alpha oscillation of sensitivity is likely to represent the periodical engagement and disengagement of sensory processing controlled by rhythmic top-down attention. For criterion, however, the situation is more complicated.

One possible explanation for the oscillation of criterion is that it reflects dynamic top-down expectation. A previous study on cats revealed strong interactions between cortical areas in the theta-alpha range (4–12 Hz) only for the presentation of known visual stimuli, not for novel or unexpected visual stimuli^44^. The phase-synchronization in the alpha band between frontal and posterior regions indicated a top-down drive important for anticipation. Another study on monkeys revealed strong synchronization of neural activities during the expectation stage before the onset of visual stimuli^45^. Recent findings on humans also suggest that top-down prior expectations periodically bias decision-making before stimulus onset in the alpha range^30,33^. By manipulating prior expectations, Sherman *et al*. revealed that the phase of occipital alpha oscillations influenced decision criterion in a visual detection task^30^. However, it is not clear whether the prior expectation is the expectation of target present/absent *per se* (choice), or the expectation of responses, because the choice-response mapping was consistent in their experiment at all times. The alpha oscillation of criterion in the current study might also reflect top-town anticipation, in line with these findings. However, in the current study, the oscillation of criterion was susceptible to manipulation of the choice-response mapping. After we changed the mapping between choices and responses, the phase of the criterion oscillation changed accordingly, suggesting that the oscillation of criterion was driven by an oscillation of motor bias. This implies that the criterion oscillation reported here reflects dynamic top-down expectation on motor response rather than expectations about grating orientation *per se*.

The other possibility is that criterion reflects fluctuation of spontaneous motor preparation, independent of sensory processing. Traditionally, motor output was viewed as the late stage of a series of processes that executes responses. However, more and more recent evidence suggests that motor-related activities play an important role in decision-making^46,47^. Motor competition between potential actions can take place before the final decision^48,49^. Moreover, perceptual decisions can be influenced by the physical resistance applied to the response^50^. By manipulating prior expectations with cues, de Lange *et al*. found a suppression of pre-stimulus low-frequency power (8–30 Hz) over motor cortex contralateral to the upcoming movement^33^. More importantly, when the cue was not informative, they still observed a lateralization of pre-stimulus low-frequency power over motor cortex related to choice bias. However, the choice and motor response were not separable in their experiment and so it is hard to clarify whether the oscillatory activities over motor areas reflected motor bias as such or the influence of perceptual expectation. Recently, Pape and Siegel designed an elegant experiment to dissociate choices from motor responses^51^. They randomly assigned the mapping between choices and responses during each trial and the cue indicating the mapping was presented after the stimulus presentation. They found beta (12–30 Hz) lateralization over motor cortex both after the choice-response cue and before the onset of stimulus. This response-predictive beta lateralization reflects the encoding of the upcoming response rather than choice content because choice and motor responses were dissociated. The oscillation of criterion we found here may reflect rhythmical motor preparation independent of sensory processing. It is possible that voluntary action can synchronize oscillations for both sensory processing (sensitivity) and spontaneous motor preparation (criterion) that contribute to late decision-making.

In summary, using the framework of SDT, we found that voluntary actions synchronize behavioral oscillations of both sensitivity and criterion, suggesting a general role in resetting the state of neural activity. The alpha oscillation of sensitivity was immune to the manipulation of choice-response contingency, suggesting it might reflect the periodic engagement and disengagement of sensory processing controlled by rhythmic top-down attention. The alpha oscillation of criterion that was susceptible to the manipulation of choice-response contingency suggests a rhythmic motor preparation, although it is not clear whether the oscillatory motor preparation is spontaneous or controlled by top-down expectation.

## Methods

### Participants

17 students (3 male) from the University of Sydney, aged 18–32 years, formed Group 1, and 14 new students (4 male, aged 19–35 years) were recruited for Group 2. They had normal or corrected-to-normal vision and normal audition. The study was carried out in accordance with the Declaration of Helsinki and was approved by the Ethics Committee of the University of Sydney. Participants gave informed consent before the experiment.

### Apparatus

The experiment was run in a dimly lit room. The ambient luminance was 2.1 cd/m^2^. Participants were seated in front of a matte white PVC screen (Epson ELP-SC21B, 1771 × 996 mm) with a viewing distance of 1.4 m. We used a PROPixx color projector (VPixx Technologies Inc.) to present visual stimuli on a casted area of 117 × 66 cm (45.4 × 26.5° of visual angle) at a resolution of 1920 × 1080 pixels at a refresh rate of 120 Hz. The projector was set to quadrant mode, resulting in a resolution of 960 × 540 pixels at a frame rate of 480 Hz when displaying images. The luminance output of the projector was linearized. Participants’ heads were maintained as stationary by using a chin-rest. We used headphones to deliver sound stimuli. Experimental programs for this study were developed with Matlab 2015a (MathWorks Inc., Natick, MA) and Psychophysics Toolbox^52,53^.

### Stimuli and experimental procedure

The task was to discriminate two possible orientations (45° clockwise or anticlockwise, i.e., 45° or −45°) of a grating embedded in noise (Fig. 1). The target was presented on a grey background (92.7 cd/m^2^). The target onset time varied across trials, ranging from 0 to 800 ms in intervals of 5 ms (160 time points). In each block, each onset time appeared once for each orientation, resulting in 320 trials in total. Each participant attended two sessions over two days, each of which consisted of three blocks. They took a short break every 64 trials.

In each block, participants were asked to maintain their fixation on a central cross (0.35° wide) during the task. They held the RESPONSEPixx (VPixx Technologies Inc.) button box with their left hands and the mouse with their right hands. Participants used their left thumb to press a button on the RESPONSEPixx box to start each trial. After the button-press, a grating embedded in noise was presented for 3 frames (6.3 ms) at one of the time points in the 0–800 ms range (Fig. 1). The grating spatial frequency was 2.5 cpd and it was embedded in additive white noise and then multiplied by Gaussian annulus window with a standard deviation of 0.3° and which peaked at 1.0° away from the central cross. The white noise was randomly generated in each trial, but its contrast was constant (30%) for all the participants. The noise was filtered so that its spatial frequency matched the spatial frequency of the grating. After presentation of the target, participants clicked a mouse with their right hand to indicate which orientation they perceived. For the participants in Group 1, they responded with left-click to indicate anticlockwise and right-click for clockwise. For the participants in Group 2, the mapping between orientation and mouse-click was reversed. Because we were more interested in accuracy than speed, we specifically asked participant to slow down when making responses and prioritise accuracy. After their response, no feedback was given and they were required to wait for at least 2 s before pressing the button to start next trial. If they started too early, they would hear a brief beep (1000 Hz, 20 ms) and they needed to wait for two more seconds to start the trial again. Before formal testing, we used an Accelerated Stochastic Approximation (ASA) procedure^54,55^ to adjust the contrast of the grating for each observer to yield 75% correct responses. This contrast value was then used for the first trial in the formal experiment. The contrast value was adjusted trial by trial with the same ASA procedure (determined by performance in preceding 30 trials) during testing to ensure that performance was always close to threshold.

### Data Analysis

The ideal stimulus onset time varied in the range of 0–800 ms in steps of 5 ms. However, inevitable delays of computer refreshing mean that the actual stimulus onset time can vary by several milliseconds. All our data analyses were conducted on the actual time of stimulus presentation. We binned the data every 15 ms (53 data points in the range of 5–800 ms). We applied signal detection theory (SDT)^25^ to analyse the data (Fig. 2). In the framework of SDT, observers’ decisions can be summarized by the sensitivity (d′) and criterion (c), with sensitivity representing observers’ ability to distinguish signals from noise and criterion indicating observers’ decision bias. Here, we analysed data based on either stimulus or response. For the stimulus-based analysis, we chose the anticlockwise orientation as the “Signal” condition to analyse our data. For each bin, we calculated the Hit rate and the False Alarm rate. The Hit rate (i.e., correct detection) is given by the percentage of ‘anticlockwise’ choices in trials containing the anticlockwise grating, and the False Alarm rate is the percentage of ‘anticlockwise’ choices in trials containing the clockwise grating. For the response-based analysis, we chose the ‘left-click’ as the “Signal” condition. The Hit rate is given by the percentage of ‘left-click’ in trials where the correct answer is ‘left-click’ (trials presenting anticlockwise grating in Group 1, and clockwise grating in Group 2). The False Alarm rate was acquired by the percentage of ‘left-click’ in trials where the correct answer was ‘right-click’ (trials presenting clockwise grating in Group 1, and anticlockwise grating in Group 2). We calculated the sensitivity (d′) and criterion (c) with following equations, where Z(HR) means the z-score transformation of hit rate, based on normal distribution and Z(FAR) means the z-score transformation of false alarm rate. For criterion c, positive values represent a bias towards clockwise orientation, and negative values represent a bias towards clockwise orientation.

$$d\text{'}=\frac{Z(HR)-Z(FAR)}{\sqrt{2}}$$

$${\rm{c}}=-\,\frac{Z(HR)+Z(FAR)}{2}$$

We pooled the data from two groups of participants together as the aggregated observer. We conducted curve fitting to evaluate the presence of oscillations for the aggregated observer. For the curve fitting, the time series of the aggregated observer were fitted with first-order Fourier series (${\rm{y}}={a}_{0}+{a}_{1}\,\cos \,2\pi ft+{b}_{1}\,\sin \,2\pi ft$, where *f* is the frequency; *a*_0_, *a*_1_, and *b*_1_ are Fourier coefficients; t is time) in the theta-alpha range (3–13 Hz). The variance of the data in each bin was estimated with 1000 bootstraps^56^. The significance of curve fitting was tested with the permutation method^57^. For each permutation, we shuffled the responses across trials for each grating orientation separately. Then we binned and fitted the permutated data with the Fourier series in the same frequency range (3–13 Hz) with *f*, *a*_0_, *a*_1_, and *b*_1_ as free parameters. The R^2^ value of the best Fourier fit to the permutated data was recorded and the permutation procedure was repeated 5000 times, resulting in a distribution of 5000 R^2^ values. The permutation test was run to assess whether the R^2^ of the best-fit of the time series was higher than the 95% of the R^2^ obtained from the permutated data.

To further examine the oscillation frequency spectrum, we also fitted the first-order Fourier series in steps of 0.5 Hz from 4–12 Hz (17 frequencies) to the time series of the aggregated data. For aggregated data, we ran permutation tests (n = 5000) to examine the significance of spectrum amplitude at each of the 17 frequencies. At each frequency under test, we fitted first-order a Fourier series with frequency fixed and all other parameters free. The permutation test was run to assess whether the amplitude of the best-fit of the time series was higher than 95% of the amplitudes obtained from the permutated data. We corrected the resulting p-values with false discovery rate (FDR) correction (10%) for multiple comparisons^34^.

For frequency spectrum in the range of 4–12 Hz in steps of 0.5 Hz (17 frequencies), we also performed a random-effect statistical test. For each participant, we binned the data every 25 ms (32 data points in the range of 2.5–800 ms). Then we fitted the first-order Fourier series (${\rm{y}}={a}_{0}+{a}_{1}\,\cos \,2\pi ft+{b}_{1}\,\sin \,2\pi ft$, where *f*is the frequency; *a*_0_, *a*_1_, and *b*_1_ are Fourier coefficients; t is time) to the time series and acquired a vector of cosine and sine components (${\rm{X}}=[{a}_{1},\,{b}_{1}]$) at each of the 17 frequencies. Hotelling’s T-square test is appropriate for multivariate test^36,58^, but it requires a multivariate normal distribution. Hence, we used permutation when using Hotelling’s T-square test^59^ to examine whether the mean of these vectors was different from *μ*_0_, [0, 0]. The Hotelling’s T-square statistic is: ${T}^{2}=n(\bar{X}-{\mu }_{0})^{\prime} {S}^{-1}(\bar{X}-{\mu }_{0})$, where *n* is the number of participants (*n* = 29); $\bar{X}$ is mean of vector, $\bar{X}=[\overline{{a}_{1}},\overline{{b}_{1}}]$); *S* is the sample covariance matrix. The permutation Hotelling’s T-square test was performed at each frequency. At the tested frequency, for each permutation, for each participant, their responses were shuffled across trials, and then we did the same data analysis (binning, SDT analysis, and fitting with the tested frequency). The T-square was calculated using the resulting 29 vectors of cosine and sine components and we used 5000 permutations to create the null-distribution of T-square. The p-value was the percentage of permutated T-squares that were greater than the T-square obtained from the real data at that frequency. The 17 p-values we obtained were then corrected for multiple comparison correction with 10% FDR.

For phase, we examined phase consistency between the individual participants with the Rayleigh test in the CircStats toolbox^35^ in the range of 4–12 Hz. The results at 17 frequencies were corrected for multiple comparisons (FDR = 10%).

## Supplementary information

Supplementary Information

## Data Availability

The datasets generated during and/or analyzed during the current study are available from the corresponding author on request.
