# Supplementary material for: Behavioural oscillations in visual orientation discrimination reveal distinct modulation rates for both sensitivity and response bias
Source: Sci Rep. 2019 Feb 4;9:1115. doi: 10.1038/s41598-018-37918-4 (PMC6362039; doi:10.1038/s41598-018-37918-4)
Supplement: Supplementary file 1 — Supplementary Information [file 41598_2018_37918_MOESM1_ESM.docx]

# Supplementary Information

# Behavioural oscillations in visual orientation discrimination reveal distinct modulation rates for both sensitivity and response bias

Huihui Zhang, Maria Concetta Morrone, David Alais


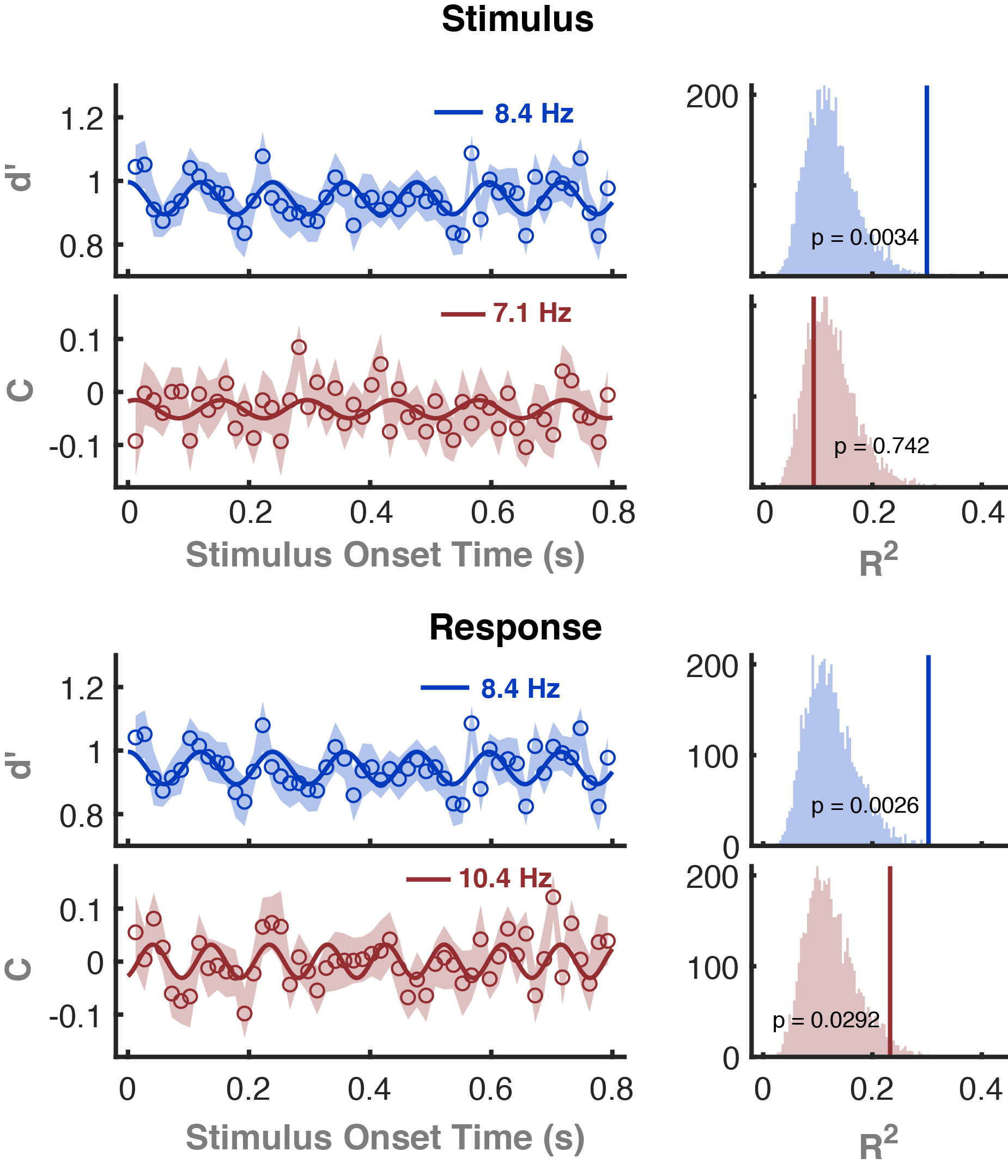


**Figure S1*.* A.** Group average performance of participants from Groups 1 and 2 (n = 29) over time on the two dependent measures (sensitivity and criterion) with anlaysis based on Stimulus (A) and on Response (B). Left column: the open symbols are the real data points, with ±1 SEM (shaded area), at intervals of 15 ms. The continuous line is the best-fitting curve from the Fourier series. Right column: results from the permutation test (n = 5000). The histograms show the distribution of R^2^-values obtained from fitting the Fourier series to the permutated data. The red and blue vertical lines show the R^2^ of the best Fourier series fitted to the real data, with its p-value labelled.
